# Whales from space dataset, an annotated satellite image dataset of whales for training machine learning models

**DOI:** 10.1038/s41597-022-01377-4

**Published:** 2022-05-27

**Authors:** Hannah C. Cubaynes, Peter T. Fretwell

**Affiliations:** 1 British Antarctic Survey, High Cross, Madingley Road, Cambridge, CB3 0ET UK; 2 Scott Polar Research Institute, University of Cambridge, Lensfield Road, Cambridge, CB2 1ER UK

**Keywords:** Conservation biology, Image processing

## Abstract

Monitoring whales in remote areas is important for their conservation; however, using traditional survey platforms (boat and plane) in such regions is logistically difficult. The use of very high-resolution satellite imagery to survey whales, particularly in remote locations, is gaining interest and momentum. However, the development of this emerging technology relies on accurate automated systems to detect whales, which are currently lacking. Such detection systems require access to an open source library containing examples of whales annotated in satellite images to train and test automatic detection systems. Here we present a dataset of 633 annotated whale objects, created by surveying 6,300 km2 of satellite imagery captured by various very high-resolution satellites (i.e. WorldView-3, WorldView-2, GeoEye-1 and Quickbird-2) in various regions across the globe (e.g. Argentina, New Zealand, South Africa, United States, Mexico). The dataset covers four different species: southern right whale (*Eubalaena australis*), humpback whale (*Megaptera novaeangliae*), fin whale (*Balaenoptera physalus*), and grey whale (*Eschrichtius robustus*).

## Abstract (summary)

**Table:** 

Measurement(s)	Whale detections in very high-resolution satellite imagery
Technology Type(s)	very high-resolution satellites and GIS software
Sample Characteristic - Organism	Megaptera novaeangliae • Balaenoptera physalus • Eschrichtius robustus • Eubalaena australis
Sample Characteristic - Location	Maui, United States • Peninsula Valdes, Argentina • Pelagos Sanctuary • Auckland Islands, New Zealand • Laguna San Ignacio, Mexico• Witsand, South Africa

## Background & Summary

Very high-resolution (VHR) satellite imagery allows us to survey regularly remote and large areas of the ocean, difficult to access by boats or planes. The interest in using VHR satellite imagery for the study of great whales (including sperm whales and baleen whales) has grown in the past years^1–5^ since Abileah^6^ and Fretwell *et al*.^7^ showed its potential. This growing interest may be linked to the improvement in the spatial resolution of satellite imagery, which increased in 2014 from 46 cm to 31 cm. This upgrade enhanced the confidence in the detection of whales in satellite imagery, as more details could be seen, such as whale-defining features (e.g. flukes).

Detecting whales in the imagery is either conducted manually^1,4,5,7^, or automatically^2,3^. A downside of the manual approach is that it is time-demanding, with manual counter often having to view hundred and sometimes thousands of square kilometres of open ocean. The development of automated approaches to detect whales by satellite would not only speed up this application, but also reduce the possibility of missing whales due to observer fatigue and standardize the procedure. Various automated approaches exist from pixel-based to artificial intelligence. Machine learning, an application of artificial intelligence, seems to be the most appropriate automated method to detect whales efficiently in satellite imagery^2,3,8,9^.

In machine learning an algorithm learns how to identify features by repeatedly testing different search parameters against a training dataset^10,11^. Concerning whales, the algorithm needs to be trained to detect the wide variety of shapes and colour characterising whales. Shapes and colour will be influenced by the type of species, the environment (e.g. various degree of turbidity), the light conditions, and the behaviours (e.g. foraging, travelling, breaching), as different behaviours will result in different postures. The larger a training dataset is, the more accurate and transferable to other satellite images the algorithm will be. At the time of writing, such a dataset does not exist or is not publicly available.

Creating a large enough dataset necessary to train algorithms to detect whales in VHR satellite imagery will require the various research groups analysing VHR satellite imagery to openly share examples of whales and non-whale objects in VHR satellite imagery, which could be facilitated by uploading such data on a central open source repository, similar to the GenBank^12^ for DNA code or OBIS-Seamap^13^ for marine wildlife observations. Ideally clipped out image chips of the whale objects would be shared as tiff files, which retains most of the characteristics of the original image. However, all VHR satellites are commercially owned, except for the Cartosat-3 owned by the government of India^14^, which means it is not possible to publicly share image chips as tiff file. Instead, image chips could be shared in a png or jepg format, which involve loosing some spectral information. If tiff files are required, georeferenced and labelled boxes encompassing the whale objects could also be shared, including information on the satellite imagery to allow anyone to ask the commercial providers for the exact imagery.

**Fig. 1:**
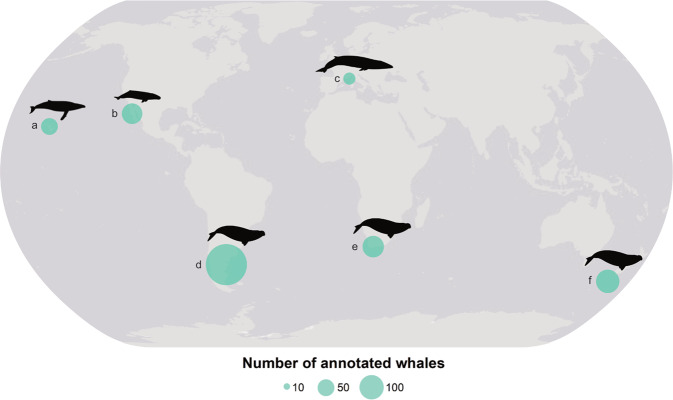
Database of annotated whales detected in satellite imagery covering different species and areas. Humpback whales were detected in Maui Nui, US (**a**); grey whales in Laguna San Ignacio, Mexico (**b**); fin whales in the Pelagos Sanctuary, France, Monaco and Italy (**c**); southern right whales were observed in three areas, off the Peninsula Valdes, Argentina (**d**); off Witsand, South Africa (**e**); and off the Auckland Islands, New Zealand (**f**). The dot size represents the number of annotated whales per location. Whale silhouettes were sourced from philopic.com (the grey and humpback whales silhouettes are from Chris Luh).

Here we present a database of whale objects found in VHR satellite imagery. It represents four different species of whales (i.e. southern right whale, *Eubalaena australis*; grey whale, *Eschrichtius robustus*; humpback whale, *Megaptera novaeangliae*; fin whale, *Balaenoptera physalus*; Fig. 1), which were manually detected in images captured by different satellites (i.e., GeoEye-1, Quickbird-2, WorldView-2, WorldView-3). We created the database by (i) first detecting whale objects manually in satellite imagery, (ii) then we classified whale objects as either “definite”, “probable” or “possible” as in Cubaynes *et al*.^1^; and (iii) finally we created georeferenced and labelled points and boxes centered around each whale object, as well as providing image chips in a png format. With this database made publicly available, we aim to initiate the creation of a central database that can be built upon.

## Methods

### Image acquisition

Twelve satellite images were used to build the database. They were acquired by different very high-resolution satellites owned by Maxar Technologies, formerly known as DigitalGlobe (Table 1). The choice of imagery was linked to other projects^1,3,7,8^ or specifically acquired to enlarge the database. Some images were selected from Maxar Technologies’ archives^15^ and others were requested to be captured during a specific time window (see “Usage note” section for advice about access to satellite images).

**Table 1:** Characteristics of the satellite imagery analysed for the presence of whales.

Location	Target Species	Satellite	Catalogue ID	Product Type and Level	Date (DD/MM/YYYY)	Max Ground Sample Distance	Bands	Area (km^2^)
Auckland Islands, New Zealand	Southern right whale (*Eubalaena australis*)	QuickBird-2	1010010005232700	Standard 2 A	12/08/2006	0.65 m	4xMULs PAN	70
Auckland Islands, New Zealand	Southern right whale	WorldView-2	103001000D6D1000	Standard 2 A	27/08/2011	0.48 m	8xMULs PAN	70
Laguna San Ignacio, Mexico	Grey whale (*Eschrichtius robustus*)	WorldView-3	104001002959ED00	Standard 2 A	20/02/2017	0.39 m	8xMULs PAN	350
Maui Nui, US	Humpback whale (*Megaptera novaeangliae*)	WorldView-3	1040010006C2B700	Standard 2 A	09/01/2015	0.36 m	8xMULs PAN	570
Pelagos, Ligurian Sea	Fin whale (*Balaenoptera physalus*)	WorldView-3	104001001E19F000; 104001001E7B8900; 104001001E020000; 104001001D325700	Standard 2 A	19/06/2016 19/06/2016 19/06/2016 26/06/2016	0.33 m 0.37 m 0.39 m 0.34 m	8xMULs PAN	4,230
Península Valdés, Argentina	Southern right whale	WorldView-2	103001001C8C0300	Standard 2 A	19/09/2012	0.56 m	4xMULs PAN	120
Península Valdés, Argentina	Southern right whale	WorldView-3	10400100032 A3700	Standard 2A	16/10/2014	0.37 m	8xMULs PAN	560
Península Valdés, Argentina	Southern right whale	WorldView-2	103001005CBC0A00	Stereo 1B	23/09/2016	0.55 m	8xMULs PAN	270
Witsand, South Africa	Southern right whale	GeoEye-1	1050410001D94500	Standard 2 A	09/08/2009	0.44 m	4xMULs PAN	60

MUL refers to multispectral imagery, which is composed of various colour bands (e.g. four or eight). PAN refers to panchromatic, which is always composed of one greyscale band.

Criteria to select the imagery were: 1) less 20% cloud cover, 2) calm sea state (i.e. no white caps and low swell), and 3) where it was known that only one species would be present at the time of image acquisition. The percentage of cloud coverage was assessed by the satellite imagery provider. We visually assessed the sea state for the presence of white caps and the level of swell. As it is currently unknown whether species could be differentiated in VHR satellite images, we selected well studied locations to ensure the presence in the imagery of only one great whale species.

### Detecting whales

The satellite images were manually scanned for the presence of whales using ArcGIS 10.4 ESRI 2017, following Cubaynes *et al*.^1^ systematic method, which involved overlaying a grid on top of the imagery and scanning one cell after the other at a scale of 1:1,500 m. Prior to scanning, the imagery was pansharpened, a process of joining the high spatial resolution of the panchromatic image (grey scale image) to the high spectral resolution of the multispectral image (colour image) to get one image of high spatial and spectral resolutions. We used the ESRI pansharpening algorithm.

Whale objects were marked with a point and were subsequently assigned a level of confidence as explained below in the “Technical Validation” section.

### Creating labelled and georeferenced points and boxes

**Fig. 2:**
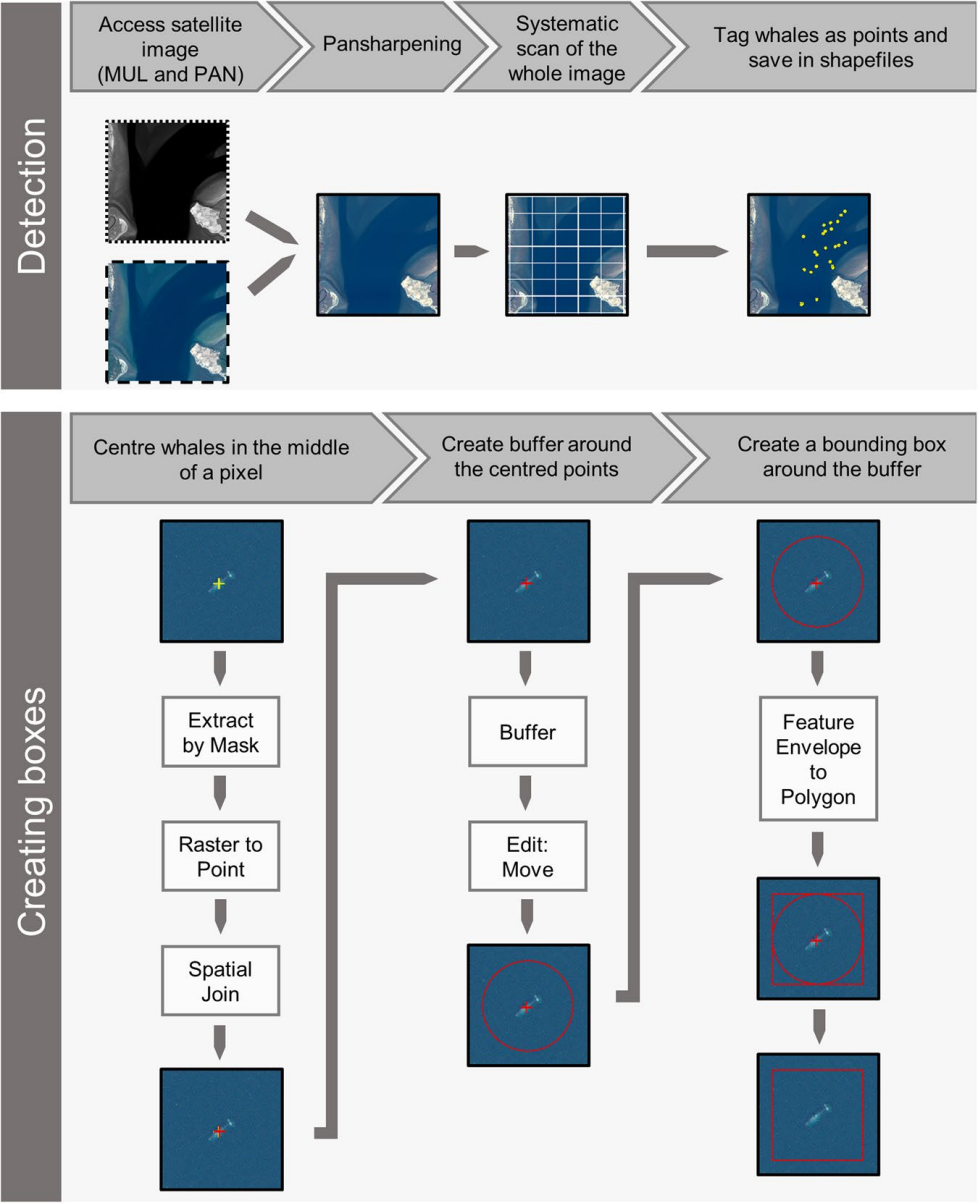
Workflow presenting the various steps to create the Whales from Space database, using ArcGIS 10.4 ESRI 2017. The multispectral image is outlined by large black dashes, the panchromatic by small black dashes and the pansharpened by a full black line. Satellite images © 2022 Maxar Technologies.

For each detected whale, a point was placed on it with associated metadata (see Data description). Boxes were created around each point indicating a whale object using ArcGIS 10.4 ESRI 2017, and following the workflow illustrated in Fig. 2. We created square boxes with a power of 2 (i.e. 128 × 128 pixels) to facilitate its use for machine learning approaches, particularly deep learning algorithms. Each whale object was represented by a point and a box (delimiting the pixels in the pansharpened image). The boxes created around the whale object were saved as one shapefile (a georeferenced file) per satellite image, as the coordinate system varied from one image to the next (Table 2), similarly for points. With the exception of the four satellite images of the Pelagos Sanctuary, for which one box and one point shapefile were created for the four images.

**Table 2:** List of shapefiles included in the dataset that represents whale objects examples in VHR satellite imagery.

File Name	Description		
	*Species*	*Image Catalogue ID*	*Location*
Box_Auckland2006_Whales_PS.shp Point_Auckland2006_Whales_PS.shp	Southern right whale	1010010005232700	Auckland Islands, New Zealand
Box_Witsand2009_Whales_PS.shp Point_Witsand2009_Whales_PS.shp	Southern right whale	1050410001D94500	Witsand, South Africa
Box_Auckland2011_Whales_PS.shp Point_Auckland2011_Whales_PS.shp	Southern right whale	103001000D6D1000	Auckland Island, New Zealand
Box_Valdes2012_Whales_PS.shp Point_Valdes2012_Whales_PS.shp	Southern right whale	103001001C8C0300	Península Valdés, Argentina
Box_Valdes2014_Whales_PS.shp Point_Valdes2014_Whales_PS.shp	Southern right whale	10400100032A3700	Península Valdés, Argentina
Box_Maui2015_Whales_PS.shp Point_Maui2015_Whales_PS.shp	Humpback whale	1040010006C2B700	Maui Nui, US
Box_Pelagos2016_Whales_PS.shp Point_Pelagos2016_Whales_PS.shp	Fin whale	104001001E19F000; 104001001E7B8900; 104001001E020000; 104001001D325700	Pelagos Sanctuary, Ligurian Sea
Box_Valdes2016_Whales_PS.shp Point_Valdes2016_Whales_PS.shp	Southern right whale	103001005CBC0A00	Península Valdés, Argentina
Box_Ignacio2017_Whales_PS.shp Point_Ignacio2017_Whales_PS.shp	Grey whale	104001002959ED00	Laguna San Ignacio, Mexico

### Creating image chips

**Fig. 3:**
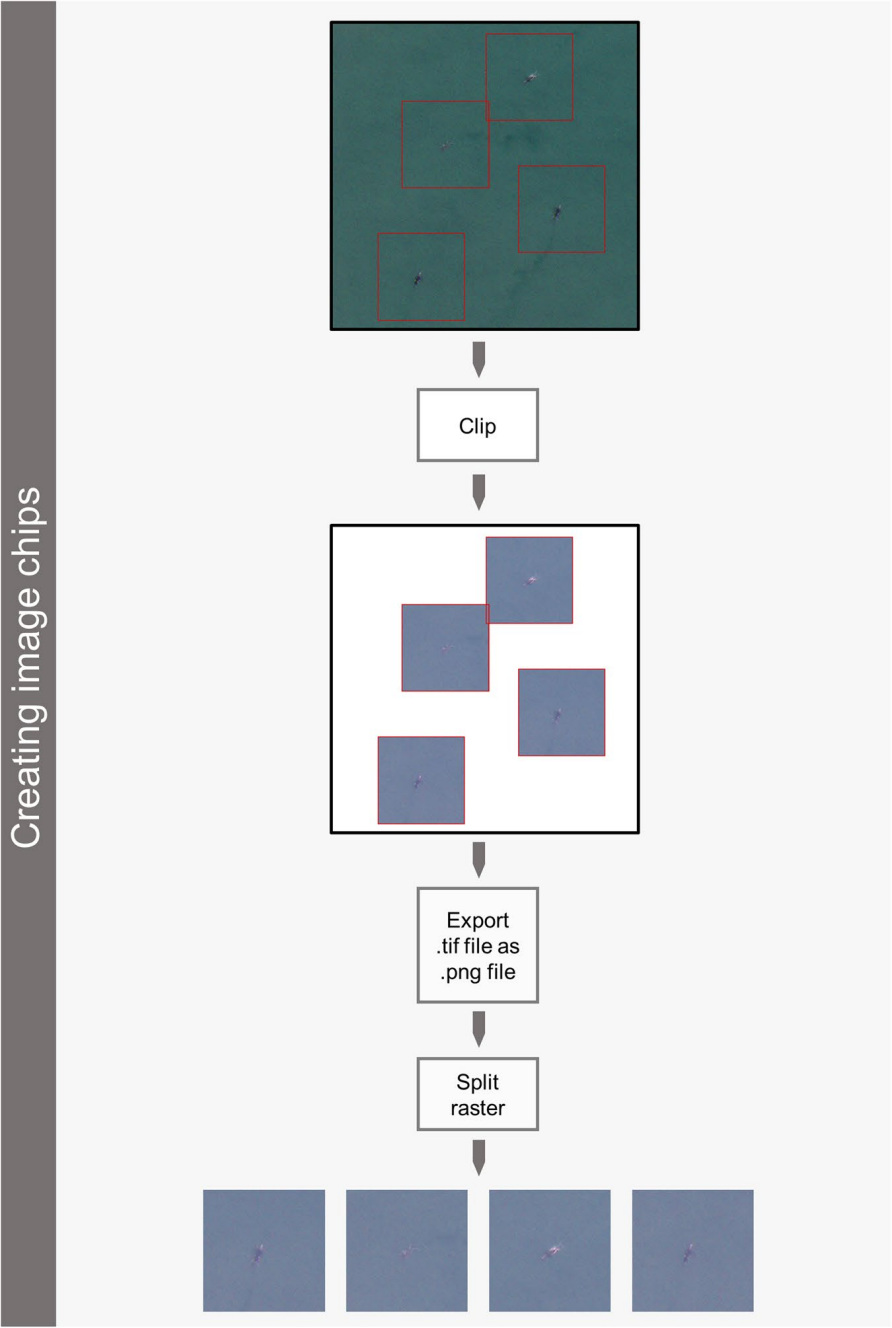
Workflow presenting the steps to create the image chips using ArcGIS 10.4 ESRI 2017 and the pansharpened image and boxes created in Fig. 2. Satellite images © 2022 Maxar Technologies.

Image chips were created using the box created in the above section, following the workflow presented in Fig. 3. Prior to creating the image chips for Valdes 2012 and 2016, the corresponding box shapefiles and satellite images had to be re-projected to WGS 1984 UTM Zone 20 S. We did the same for Auckland 2006 and 2011, using WGS 1894 UTM Zone 58 S. The file name of the image chips corresponds to the box ID of the respective boxes, allowing to find the associated data within the corresponding box shapefiles that defines the specific raw satellite image that the image chip came from.

### Future updates of the datasets

As we acquire and analyse more satellite imagery, we aim to annually update the Whales from Space dataset. The updates will be available under the Whales from Space dataset deposited on the NERC Polar Data Centre repository^16^,^17^ to ensure consistency and long-term public availability of the data.

## Data Records

**Fig. 4:**
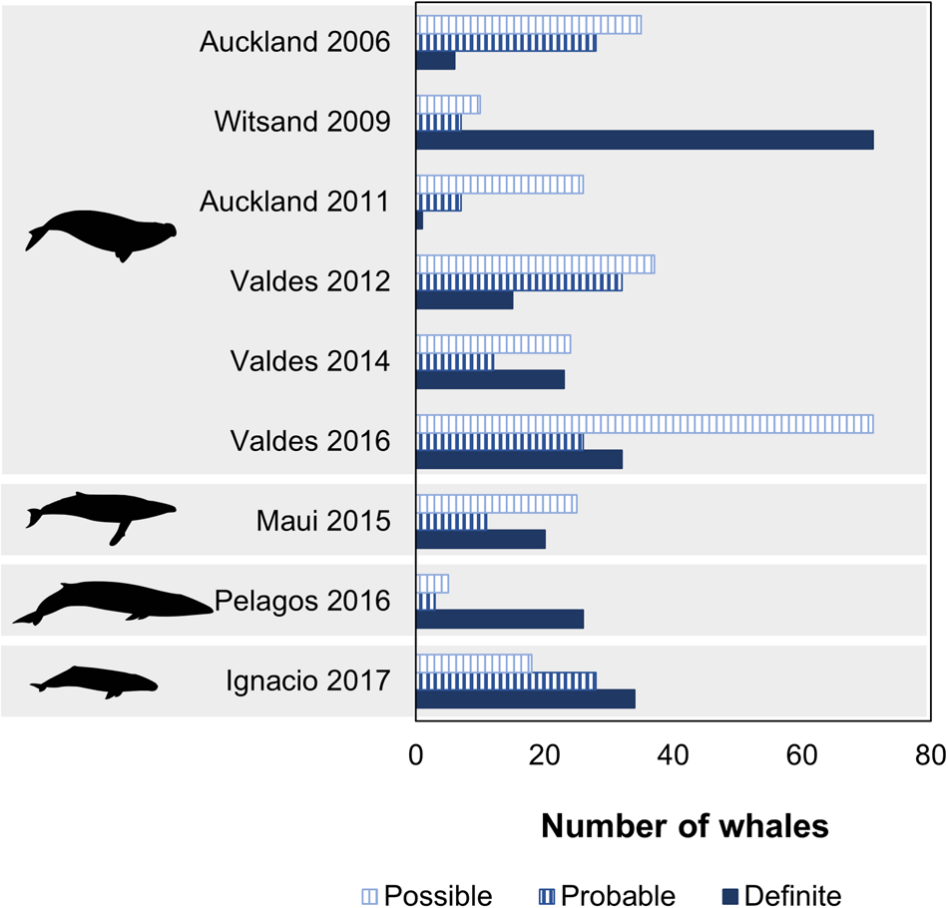
Proportion of whale objects included in the database per species (top to bottom: southern right whale, humpback whale, fin whale and grey whale) and per certainty categories (“definite”, “probable”, and “possible”). The proportion is given separately for each satellite image analysed in this study (Table 1).

The “Whales from space dataset” is available on the NERC UK Polar Data Centre repository and separated in two sub-datasets: a dataset that contains the whale annotations (box and point shapefiles with associated csv files) named “Whales from space dataset: Box and point shapefiles”^16^; and a dataset with the image chips named “Whales from space dataset: Image chips”^17^. The “Whales from space dataset: Box and point shapefiles” dataset can be accessed on the NERC UK Polar Data Centre directly using the DOI link (10.5285/C1AFE32C-493C-4DC7-AF9F-649593B97B2C). This dataset contains nine shapefiles with boxes centered on each whale and nine point shapefiles marking each individual detected whales (Table 2) totalling 633 annotated whale objects (Table 3 and Fig. 4). This dataset also includes four csv files: 1) a csv file joining all the attribute tables linked to every box and point shapefiles for whale objects (WhaleFromSpaceDB_Whales.csv); 2) a second csv file explaining each column of the first csv file (WhaleFromSpace_Guidance.csv); and 3) two other csv files describe the naming of each box (WhaleFromSpaceDB_BoxNaming.csv) and each point (WhaleFromSpaceDB_PointNaming.csv).

**Table 3:** Summary of the number of whale objects counted in the imagery.

Location and year	Definite whales	Probable whales	Possible whales	Total number of whales
Auckland 2006	6	28	35	69
Witsand 2009	71	7	11	88
Auckland 2011	1	7	26	34
Valdés 2012	15	32	37	84
Valdés 2014	23	12	24	59
Maui 2015	20	11	25	56
Pelagos 2016	26	3	5	34
Valdés 2016	32	26	71	129
Ignacio 2017	34	28	18	80
**Total**	**228**	**154**	**252**	**633**

See Table 1 for more details about the satellite imagery.

The “Whales from space dataset: Image chip” comprises of the 633 annotated whale objects as image chips. To fulfil the End User Licence Agreement with Maxar Technologies^18^, these image chips are shared in a png format, and access to the dataset is available upon request from the NERC UK Polar Data Centre that can be contacted at PDCServiceDesk@bas.ac.uk. Data access requires user name and email address, which will be shared with Maxar Technologies. Anyone using any of the image chips is also required to attribute the images properly (See Usage Notes).

Each box and point has metadata associated to it, which is included in the attribute table associated to the specific shapefile. It contains information about the detected whale: certainty level (i.e. “definite”, “probable”, “possible”) derived from the classification score assessed based on various criteria (i.e. body length, body width, body shape, body colour, flukeprint, blow, contour, wake, after-breach, defecation, other disturbance, fluke, flipper, head callosities and mudtrail) following Cubaynes *et al*.^1^ method, most likely species, and potential other species. For each annotated whale, we also provide information about the imagery analysed: the location, latitude and longitude (in decimal degrees and recorded using the same geographic coordinate system and projection as the satellite imagery), imagery ID, imagery date, type of satellite, spatial resolution, number of multispectral bands, product level and type (e.g. Standard2A). The size of each boxes was also specified in terms of pixels.

## Technical Validation

### Certainty of whale identification

Ground truthing, the process of verifying on the ground what is observed in a satellite image^19^, is not possible when attempting to detect a mobile object, such as whales, because whales visible in the imagery will have moved by the time the imagery is received by the customer for analysis, which can take between six hours up to a couple days. Alternatives have been tried, such as timing the collection of satellite image with a boat or aerial survey^20,21^. However, it is difficult to synchronise the acquisition of a satellite image with such surveys, due to several factors; including competing tasking where satellite image orders for defence and disaster relief take priority over other orders. This is currently relevant as only one very high-resolution satellite can acquire 30 cm resolution imagery. The presence of clouds is also a limiting factor, as it will prevent the detection of whales in satellite images but not impact the detection capabilities from a boat survey^20,21^. There has also been an attempt to match whales tagged with tracking devices to those observed in an imagery, but the low accuracy of the coordinates provided by the tracking devices fixed on whales did not permit this matching^8^. With this dataset, to assess our confidence whether the object observed was a whale, 1) we analysed images of well surveyed areas, where only one species was recorded at a specific time^1^; and 2) we have established a certainty level reflecting our confidence in the detection. As whales will not always be at the sea surface and as light gets attenuated with increasing depth, whales below the surface will not be as visible as those near the surface, for which characteristic-whale features can be observed (e.g. fluke, flipper). Recognising that some whale objects will be easier to detect than others, we created three levels of confidence (i.e. definite, probable, and possible). The certainty level was assigned based on a combination of criteria^1^. We recommend that only the whales with a “definite” certainty level be used to train automated detection systems.

### Species differentiation

As species differentiation has not been tested when analysing satellite images, we reference the most likely species in this database. The most likely species was assigned based on the scientific literature, hence our decision to acquire images of specific areas when only one large whale species was expected to be present^1^.

## Usage Notes

### Correct attribution for satellite images

Anyone using any of the image chips is required to attribute the image chips as follow: “Satellite image © 2022 Maxar Technologies”.

### Advice on getting access to satellite images

All the satellite images that we have used to build the dataset were provided by Maxar Technologies (formerly DigitalGlobe). We recommend contacting Maxar Technologies national office to enquire about acquisition and cost, as pricing is conducted on a user case scenario. To ensure you acquire the same satellite images we have created the boxes for, we have provided the Catalogue ID in Table 1. All the images we have used are now considered archival and accessible at a lower cost. There are different types and levels for a same satellite image and we recommend acquiring the satellite images with the same product level and type, as specified in Table 1. Acquiring a different product level or type may shift the image, meaning the whale-object boxes will not be centred on the whales they were created for.

## Data Availability

We used ArcGIS 10.4 ESRI 2017 to analyse the satellite images and create the boxes. ArcGIS 10.6 ESRI 2017 can also be used. Various pansharpening algorithm exists^22^. As we have used the ESRI pansharpening algorithm, we recommend using this one. The Gram-Schmidt is often preferred when monitoring wildlife from space^23^; however, we have found that sometimes it may shift the pansharpened image compared to the panchromatic and multispectral images. Therefore, if a pansharpening algorithm other than ESRI is used, we recommend testing that it does not shift the image or to be aware of by how many pixels it has shifted the image.
