# Author Correction: Whales from space dataset, an annotated satellite image dataset of whales for training machine learning models

**DOI:** 10.1038/s41597-022-01472-6

**Published:** 2022-06-17

**Authors:** Hannah C. Cubaynes, Peter T. Fretwell

**Affiliations:** 1 British Antarctic Survey, High Cross, Madingley Road, Cambridge, CB3 0ET UK; 2 Scott Polar Research Institute, University of Cambridge, Lensfield Road, Cambridge, CB2 1ER UK

**Keywords:** Conservation biology, Image processing, Conservation biology, Image processing

Correction to: *Scientific Data* 10.1038/s41597-022-01377-4, published online 27 May 2022

The original version of this Data Descriptor incorrectly referred to the southern whale as Eubalaena galcialis instead of Eubalaena australis in the Abstract and Table 1. This has now been corrected in the PDF and HTML versions of the Article as well as the associated metadata.

